# Fabrication of Highly Conductive Silver-Coated Aluminum Microspheres Based on Poly(catechol/polyamine) Surface Modification

**DOI:** 10.3390/polym14132727

**Published:** 2022-07-03

**Authors:** Mingzheng Hao, Lei Li, Xiaoming Shao, Ming Tian, Hua Zou, Liqun Zhang, Wencai Wang

**Affiliations:** 1 The Department of Materials Engineering, Taiyuan Institute of Technology, Taiyuan 030008, China; haomz@tit.edu.cn; 2 Key Laboratory of Beijing City on Preparation and Processing of Novel Polymer Materials, Beijing University of Chemical Technology, Beijing 100029, China; q1722506038@2980.com (L.L.); bb48westvirginia@163.com (X.S.); tianm@mail.buct.edu.cn (M.T.); zouhua@mail.buct.edu.cn (H.Z.); zhanglq@mail.buct.edu.cn (L.Z.); 3 Engineering Research Center of Elastomer Materials on Energy Conservation and Resources, Ministry of Education, Beijing University of Chemical Technology, Beijing 100029, China

**Keywords:** aluminum, silver-plating, surface modification, electrical conductivity, electromagnetic interference shielding

## Abstract

A novel and cost-effective method for the fabrication of highly conductive Al/Ag core-shell structured microspheres was proposed and investigated. The oxidative co-deposition of catechol and polyamine was firstly performed to modify the surface of the aluminum microsphere. Then, a two-step electroless plating was conducted to fabricate the Al/Ag microspheres. During the first step of the electroless plating process, the surface of the aluminum microsphere was deposited with silver nanoparticle seeds using n-octylamine and ethylene glycol. Then, during the second step of the electroless plating process, silver particles grew evenly to form a compact silver shell on the surface of aluminum via a silver mirror reaction. According to the scanning electron microscope and energy dispersive X-ray results, a compact and continuous silver layer was successfully generated on the surface of the aluminum. The valence of the sliver on the surface of the aluminum was confirmed to be zero, based on the X-ray photoelectron spectrometer and X-ray diffractometer analyses. As a result, the as-prepared Al/Ag microspheres exhibited a high conductivity of 10,000 S/cm. The Al/Ag/MVQ composite demonstrated low electrical resistivity of 0.0039 Ω·cm and great electromagnetic interference shielding effectiveness at more than 70 dB against the X-band, and this result suggests that the as-prepared composite is a promising conductive and electromagnetic shielding material.

## 1. Introduction

In recent years, electromagnetic interference (EMI) has caused significant issues, such as the malfunctioning of sensitive electronic components, divulging military secrets, and harming human health [1]. Thus, to mitigate these issues, there has been an escalating interest in exploring effective electromagnetic shielding materials for EMI [2]. Currently, electroconductive materials have been commonly used in electromagnetic shielding applications due to their ability to reflect electromagnetic waves. Electroconductive materials such as metals, e.g., copper or silver powder [3]; carbon materials, e.g., carbon nanotube or graphene [4]; polymers, e.g., polypyrrole or polyaniline [5]; and core-shell particles, e.g., silver-coated cenosphere or carbonyl nickel [6,7], have been reported. Even though these materials have shown to be promising electromagnetic shielding materials, each of these materials possesses certain disadvantages that can ultimately hinder their mass adoption during commercialization. For instance, metals possess a high density and they are costly; carbon materials are difficult to disperse in the matrix; and electroconductive polymers are expensive and they possess poor mechanical properties. Among these electroconductive materials, core-shell particles are particularly attractive due to their light-weight property, low cost, high conductivity, and ease of processing, which have triggered intense research interests in recent years.

Silver-coated aluminum microspheres have attracted keen interest as a key promising electromagnetic shielding material due to the high conductivity of silver, low cost of aluminum, high ductility, and low density [8,9]. Various strategies have been developed to metalize the surface of the microsphere, such as the magnetron sputtering method [10], chemical vapor deposition [11], electrochemical deposition [12], and electroless plating [13]. Among these, electroless plating is commonly employed in industrial applications due to its low cost, use of simple equipment, unrestricted substrates, and uniform coating. Despite these advantages, it is more challenging to plate silver on aluminum microspheres when compared to other materials. Aluminum powder can easily participate in the electroless silver-plating process, which leads to the decomposition of silver nitrate [14]. Moreover, the adhesion of silver onto aluminum is poor due to the lack of active functional groups on the surface of aluminum. Thus, to prevent the participation of aluminum powder in the reaction and to enhance the adhesion strength between silver and aluminum, it is highly necessary to perform surface pretreatment or modification. Wang et al. [14] prepared Al/Ag particles via a traditional sensitization–activation approach, which was limited by several disadvantages, such as the need for tedious processes, noxiousness, and expensive agents. Wang et al. [15] also prepared highly conductive Al/Ag microspheres via surface-thiolated functionalization. However, the thiol compounds used in this method are usually toxic, and there are VOC emissions during the surface modification process. Therefore, a simple, versatile, and green pretreatment or modification method to prepare Ag-coated Al microsphere is highly necessary.

Poly(dopamine) can deposit onto various organic and inorganic materials via the self-polymerization of dopamine in an aqueous alkaline solution under room temperature [16,17,18]. Besides, depositing a poly(dopamine) layer on the surface of the material can better prepare the surface for subsequent silver-plating [19,20]. Poly(dopamine) possesses catechol (with metal-binding ability) and nitrogen-containing groups [21,22,23], and it has been used in the deposition of compact metal coatings on various substrates, e.g., silica microspheres [24] and poly-meta-phenyleneisophthalamide fibers [25], by electroless plating. However, dopamine is costly, and its low formation rate can impede its extensive application. Motivated by the benefits of dopamine, Wang et al. [26] used a lost-cost catechol and polyamine to modify the polypropylene separator, since catechol and amine group are the two key components of dopamine. New insights were provided in their work, whereby they mimicked the mussel adhesion using low-cost chemicals. However, to the best of our knowledge, the surface modification of aluminum by catechol and polyamine for surface metallization has not yet been reported.

Herein, silver-coated aluminum microspheres were prepared by functionalizing the surface of aluminum with poly(catechol-polyamine) (PCPA), and then, electroless silver-plating. The first step was to modify the aluminum microspheres with catechol and polyamine. This led to the formation of a PCPA layer on the surface of aluminum microspheres via copolymerization and the oxidative co-deposition of catechol and polyamine in an alkaline solution. Then, the second step was to employ a two-step electroless plating process to fabricate the silver-coated aluminum microspheres. Finally, a highly conductive rubber was prepared by mixing the as-prepared Al/Ag microspheres with silicone rubber.

## 2. Experimental Section

### 2.1. Materials

An aluminum microsphere with an average particle size of 15 μm was purchased from Changsha Tianjiu Metal Material Co, Ltd. (Changsha, Hunan Province, China). Catechol, diethylenetriamine (DETA), tris(hydroxymethyl)-aminomethane (Tris), polyvinyl pyrrolidone (PVP), silver nitrate, glucose, n-octylamine, ammonia, ethanediol, and potassium sodium tartrate were purchased from Beijing Chemical Plant, Beijing, China. Methyl-vinyl silicone rubber (MVQ) was provided by the Zhonghao Chenguang Research Institute of Chemical Industry, Beijing, China. No further purification was conducted for all the purchased solvents and reagents, and they were used in the experiment as received.

### 2.2. Preparation of PCPA Modified Aluminum Microspheres

Aluminum microspheres (4 g) were immersed into 100 mL Tris-HCl buffer solution (pH = 9.0, 10 mM) consisting of catechol (10 mM) and DETA (10 mM), and it was stirred for 4 h, filtrated to separate, and then dried at 60 °C for 6 h. The as-obtained microspheres are represented with Al/PCPA.

### 2.3. Electroless Plating of Silver on the Surface of Al/PCPA

Firstly, 2 g Al/PCPA microspheres were dispersed in 50 mL ethylene glycol consisting of 10 mg PVP. Subsequently, 50 mL AgNO_3_ (17.5 mM) aqueous solution was added into the Al/PCPA dispersion, and they were thoroughly mixed. N-octylamine (830 µL) was rapidly added into the above dispersion, and then it was stirred for 60 min under room temperature, filtrated to separate, and then dried at 60 °C for 6 h. The as-prepared microspheres are denoted as Al/PCPA/Ag NPs.

Secondly, to prepare the silver-plating solution, ammonia was added into 100 mL AgNO_3_ (10 g/L) dropwise until a clear solution was obtained. After which, the pH of the solution was altered to 11 using sodium hydroxide. PVP (0.05 g) was then introduced to the silver-plating solution, and it was subjected to magnetic stirring for 5 min. Al/PCPA microspheres (2 g) were immersed into the as-prepared solution with 0.5 mL ethanol (introduced as a stabilizer), and it was magnetically stirred for 25 min. A reducing agent, i.e., 100 mL glucose (20 g/L) solution, was introduced dropwise to the mixture, and it was stirred for 60 min under room temperature, filtrated to separate, and then dried at 60 °C for 6 h. The as-prepared microspheres are denoted as Al/PCPA/Ag.

For comparison, the same silver-plating process was performed on pristine aluminum microspheres without PCPA modification, and the as-prepared microspheres are denoted as Al/Ag.

### 2.4. Preparation of Al/PCPA/Ag Filled Silicone Rubber

The formulation of the Al/PCPA/Ag microspheres/rubber composite is shown in Table 1. A 6-inch two-roll mill was used to mix the Al/PCPA/Ag microspheres with MVQ and vulcanizing agent, i.e., 2,5-Dimethyl-2,5-di(tert-butylperoxy)hexane. After which, the rubber composite was pre-formed to sheet material. Subsequently, a vulcanization process (first stage) was conducted by performing vulcanizing press at 170 °C and 10 MPa for t_90_ times. The second stage of the vulcanization process was then conducted on the rubber composites in an oven at 200 °C for 2 h. After the vulcanization process, the vulcanized rubber composites were left alone for 8 h prior to the characterization tests. The as-prepared samples are represented with Al/PCPA/Ag/MVQ.

### 2.5. Characterizations

An ESCALAB 250 X-ray photoelectron spectrometer (XPS, Thermo Electron Corporation, Waltham, MA, USA) with Al Kα X-ray source (1486.6 eV) was used to analyze the chemical compositions of the samples. During the preparation stage, the powder sample was pressed into a pellet. Then, a double-sided adhesive tape was used to mount the pellet onto a standard sample stud. The reference used in XPS analyses was the C 1s hydrocarbon peak (284.6 eV) to off-set the surface-charging effects.

To determine the crystalline structure of the sample, D/Max2500VB2+/PC powder X-ray diffractometer (XRD, Rigaku, Mitsuaki, Japan), with Cu Kα radiation (wavelength of 1.54056 Å), was used. In the measurement, diffraction patterns with a 2θ range of 5−90° were recorded using the reflection mode.

To determine the morphologies and elemental composition of the surface of the sample, an S-4800 scanning electron microscope (SEM, Thermo Electron Corporation, Waltham, MA, USA) furnished with an energy-dispersive X-ray (EDX, Thermo Electron Corporation, Waltham, MA, USA) detector was employed. During the sample preparation, a double-sided adhesive tape was used to fix the sample onto the sample stud. Then, a thin platinum layer was sputtered onto the surface of the sample. Accelerating voltages of 20 kV and 200 kV were used for the SEM measurements and EDX test, respectively.

Four-point probe (RTS-8, Guangzhou, China) tests were carried out to analyze the electrical conductivity of the as-obtained sample. The radius of the tip was 0.004 in, and the spacing between each tip was 0.05 in. Before the measurements, the sample was pressed into a thin disc using a tablet press under 10 MPa.

The elongation at break and tensile strength of the rubber composite were recorded using a SANS EMT2000-B universal testing machine (Shenzhen, China). To prepare the sample for the test, the molded Al/PCPA/Ag/MVQ sheet was cut into a dumbbell-shaped rubber sample based on ASTM412. A crosshead speed of 500 mm/min was used for the tensile tests. ASTM2240 was used for the measurement of the shore A hardness of the material.

## 3. Results and Discussion

**Scheme 1:**
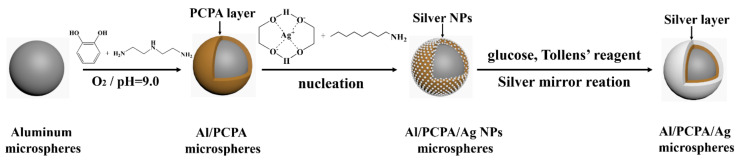
Schematic diagram of the preparation steps for the fabrication of silver-coated aluminum microspheres.

Scheme 1 shows the preparation steps for the fabrication of silver-coated aluminum microspheres. The aluminum microspheres were firstly modified with catechol and polyamine via oxidative co-deposition. Subsequently, the modified aluminum microspheres were subjected to a two-step electroless plating process. Firstly, ethylene glycol (EG) was used as a solvent to increase the reduction potential of Ag^+^ by forming an Ag/EG complex [27]. As a result, Ag^+^ ions could be easily reduced by n-octylamine to silver nanoparticles. As the catechol group in PCPA possessed metal-binding ability, silver nanoparticles could then be deposited on the surface of aluminum microsphere. Subsequently, these silver nuclei on the surface of the aluminum microsphere provided nucleation sites for the growth of silver during the next step of the electroless plating process. During the second step, [Ag(NH_3_)_2_]^+^ was reduced by glucose to silver particles. After which, the silver particles were able to grow evenly into a compact and continuous silver shell on the surface of the aluminum microspheres.

### 3.1. Surface Modification of Aluminum Microsphere with PCPA

**Scheme 2:**
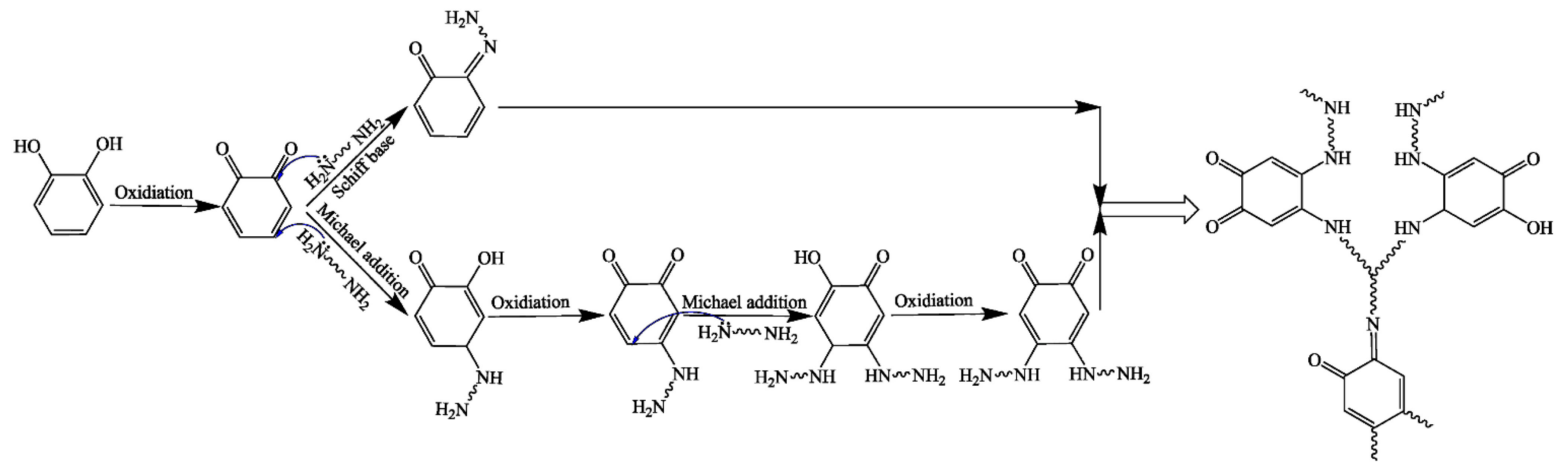
The possible reaction mechanism between catechol and polyamine.

Scheme 2 shows the possible reaction mechanism between catechol and polyamine. Firstly, catechol was oxidized to ortho-benzoquinone under alkaline conditions. The generated o-benzoquinone with active α-β-unsaturated carbonyl could subsequently react easily with the nucleophile. Two pathways occur during the subsequent reaction, i.e., Michael addition and Schiff base reaction [28]. During the Michael addition, the nitrogen atom with a lone electron pair in polyamine can attack the carbon atom at position 4 of o-benzoquinone. The addition product is then oxidized to 4-N-substituted-1, 2-benzoquinone, and it continuously reacts with polyamine via Michael addition to form 4, 5-N, N-substituted-1, 2-benzoquinone [29]. On the other side, the nitrogen atom with a lone pair of electrons attacks the carbon atom at position 2 of o-benzoquinone via Schiff base reaction to generate 2-N-substituted-1, 2-benzoquinone [30]. Finally, these two products crosslink to form poly(catechol-polyamine) (PCPA).

**Figure 1:**
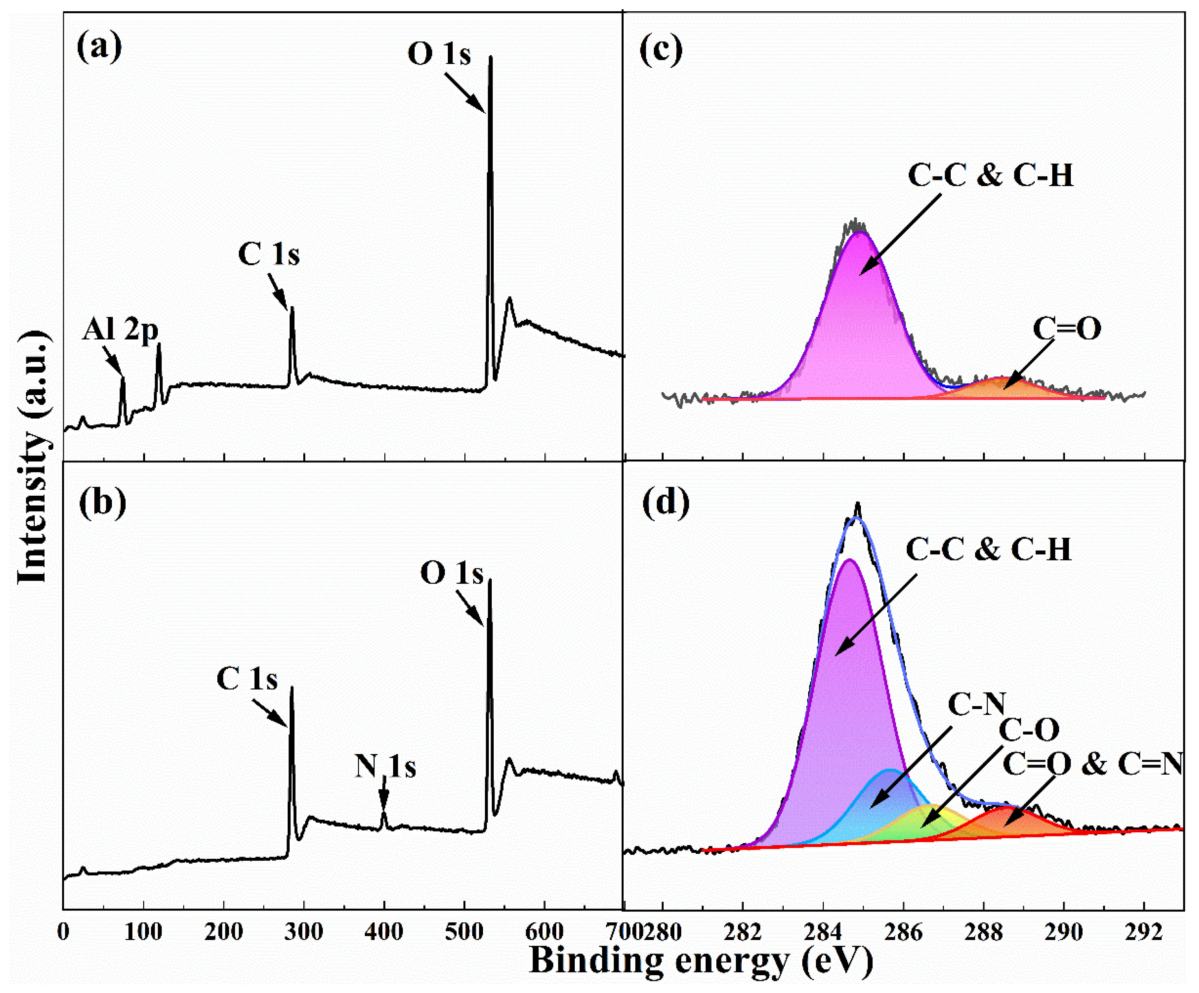
XPS survey scan spectra of (**a**) pristine aluminum and (**b**) Al/PCPA microsphere, and C1s spectra of (**c**) pristine aluminum and (**d**) Al/PCPA microsphere.

**Figure 2:**
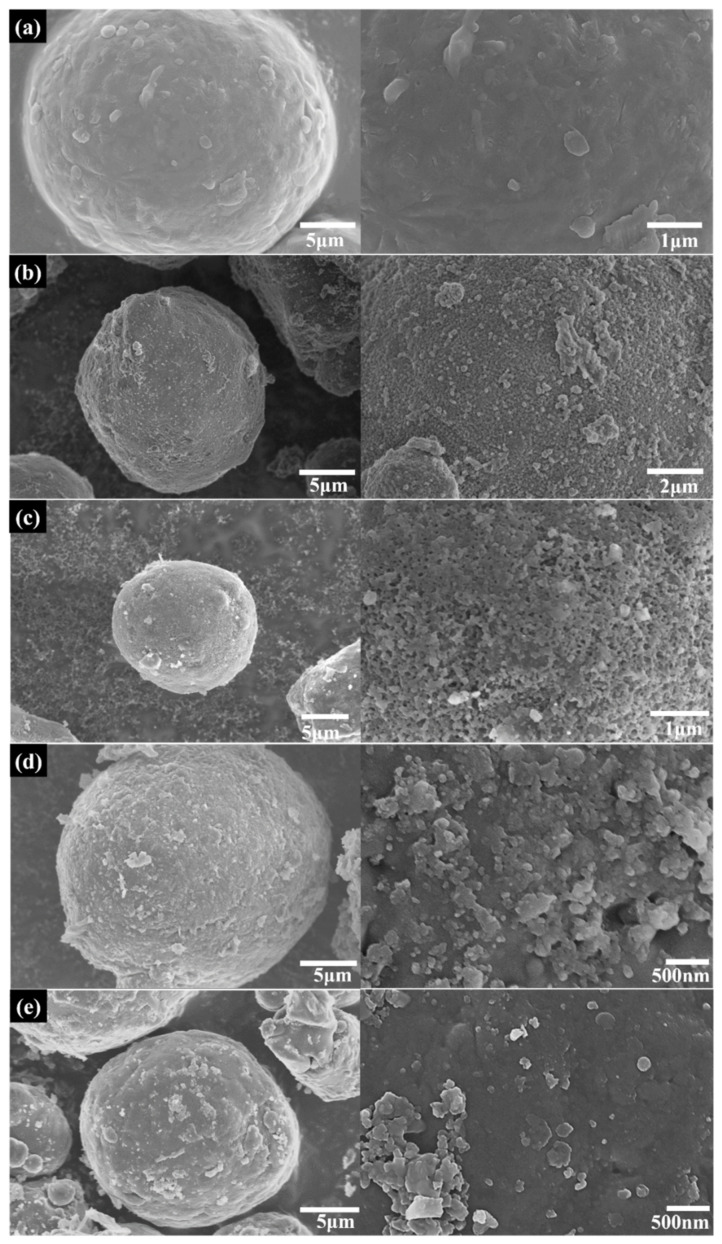
SEM images of (**a**) Al microsphere, and Al/PCPA microspheres with catechol to polyamine molar ratios of (**b**) 3:1, (**c**) 2:1, (**d**) 1:1, and (**e**) 1:3.

The chemical composition at the surface of the sample was analyzed using XPS. Figure 1 presents the XPS survey scans and C1s spectra of pristine aluminum and Al/PCPA. Elemental content percentage and carbon functionalities content percentage of pristine aluminum and Al/PCPA are shown in Table 2 and Table 3. The XPS survey scan of pristine aluminum (Figure 1a) reveals Al 2p, C1s, and O1s peaks. Interestingly, for the XPS survey scan of Al/PCPA, a new peak N1s (with 4.4 At%) located at a binding energy (BE) of 399.8 eV can be observed (Figure 1b), which may be attributed to the presence of polyamine in PCPA. In addition, the near-disappearance of the Al 2p peak (1.41 At%) and the increased percentage (30.8 At% to 57.36 At%) of C1s peak for Al/PCPA (Figure 1b) further suggest the successful deposition of PCPA on the surface of the aluminum microsphere. As shown in Figure 1c, C1s XPS core-level spectrum of pristine aluminum can be deconvoluted into two peaks, i.e., C-C/C-H (284.6 eV) and C=O (288.5 eV). These two peaks may be due to the pollution to the sample by the environment during the sample preparation process. According to Figure 2d, C1s XPS core-level spectrum of Al/PCPA microspheres reveals four peak components, i.e., C-C/C-H (284.6 eV), C-N (285.6 eV), C-O (286.6 eV), and C=O/C=N (288.5 eV) [28]. The presence of C=O in the sample is due to the quinone formed from the oxidation of catechol, whereas C=N species is attributed to the Schiff base. Thus, based on the XPS result, the polymerization mechanism of catechol and polyamine shown in Scheme 2 can be verified, and this also indicates the successful deposition of PCPA on the surface of aluminum microspheres.

SEM was employed to observe the surface morphologies of the samples. Figure 2a shows the SEM image of the pristine aluminum microsphere, whereas the SEM images of Al/PCPA microspheres are presented in Figure 2b–e. According to Figure 2a, the pristine aluminum microsphere possessed a relatively smooth surface. However, after performing surface modification to the aluminum microsphere, a rough surface could be observed (Figure 2b). Such a result indicates that the surface of aluminum microspheres was deposited with PCPA. At a catechol to polyamine molar ratio of 3:1, the formed PCPA layer was uniform and compact. As the amount of catechol decreased, the formed PCPA layer on the surface of the aluminum microsphere was incomplete and rugged. This observation may be due to the need for the adjacent hydroxyl group in catechol to help with the adhesion between the PCPA layer and aluminum [31]. Consequently, with a higher catechol concentration (to some extent), a more complete PCPA layer could be obtained. Conversely, excessive polyamine may facilitate the stacking and aggregation of oligomers. Figure 2b shows a more uniform PCPA layer than others. It is worth noting that a slightly rough surface can facilitate the interlocking of aluminum with silver nanoparticles during the electroless plating process. Thus, the optimal catechol and polyamine molar ratio used in the preparation process was set as 3:1.

### 3.2. Silver Electroless Plating on the Surface of Al/PCPA

Abundant silver nuclei on the surface of the microsphere are favorable towards the generation of a uniform and compact silver shell. To achieve this, a two-step electroless plating method was developed in this work to prepare the silver-coated aluminum microsphere. During the first step of the electroless plating method, EG was used as a solvent to generate an Ag/EG complex via internal hydrogen bonding [32]. Due to the use of EG, there is a significant increase in the reduction potential of Ag^+^ ions. This can result in the facile reduction of Ag^+^ ion by octylamine (OA), whereby the reduction of Ag^+^ ion to Ag^0^ by OA proceeds via a single electron transfer. This process results in the formation of a radical cation, and it undergoes radical disproportionation to form imine and nitrile groups [33]. As a result of the metal-binding ability of catechol and amino group in PCPA, silver nanoparticles can then be deposited on the surface of Al/PCPA. These silver particles then can act as nucleation sites for the subsequent electroless plating process. In the second step, silver nanoparticles grow on the nuclei via silver mirror reaction to generate a compact and uniform silver layer on the surface of the microsphere.

**Figure 3:**
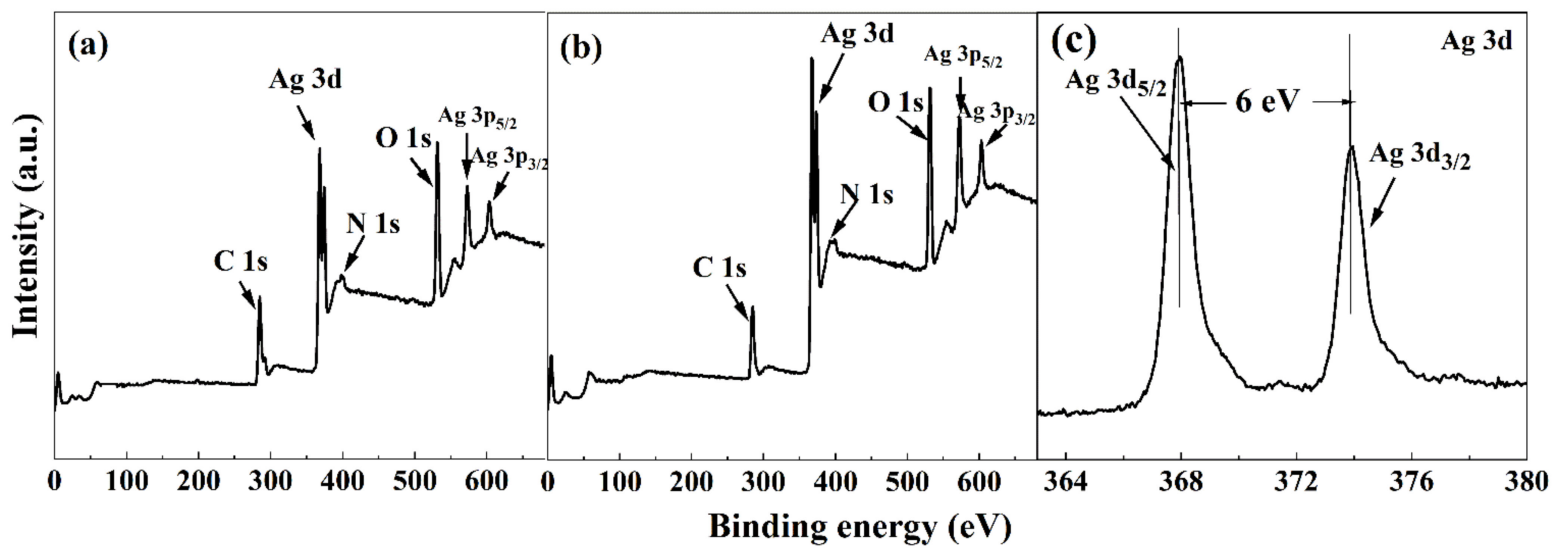
XPS survey scan spectra of (**a**) Al/PCPA/Ag NPs, (**b**) Al/PCPA/Ag, and (**c**) Ag 3d core-level spectrum of Al/PCPA/Ag microspheres.

XPS was employed to analyze the chemical state of silver on the aluminum microsphere. The XPS survey scan spectra of Al/PCPA/Ag NPs and Al/PCPA/Ag microspheres are presented in Figure 3a,b, respectively. The Ag 3d peak located at 370 eV was detected for both Al/PCPA/Ag NPs and Al/PCPA/Ag microspheres, which suggests the formation of silver nanoparticles on the surface of Al/PCPA microspheres. It is worth noting that the atomic percentage content of Ag 3d peak in Figure 3b is 9.34%, which is higher than 7.14% in Figure 3a, which suggests that more silver particles were formed on the surface of Al/PCPA/Ag microspheres as compared to that on Al/PCPA/Ag NPs. According to Figure 3c, Ag 3d core-level spectrum comprises two peaks located at 374.0 eV and 368.0 eV, which correspond to Ag 3d_3/2_ and Ag 3d_5/2_, respectively. It should be noted that these two peaks correspond to Ag^0^ species, which further confirms the existence of metallic Ag in the sample.

**Figure 4:**
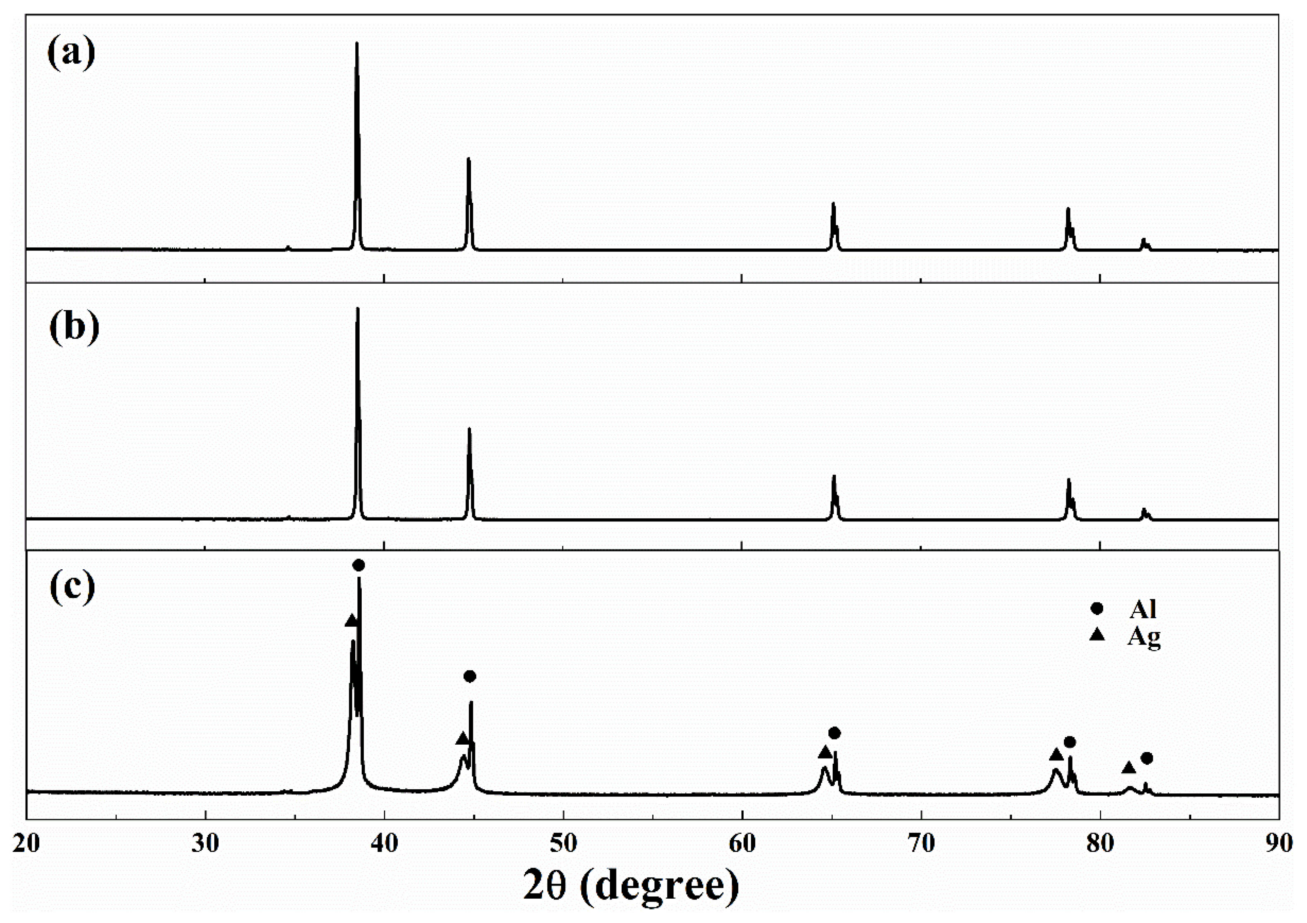
XRD spectra of (**a**) pristine aluminum, (**b**) Al/PCPA, and (**c**) Al/PCPA/Ag microspheres.

The crystal structures of the samples were investigated using XRD. Figure 4 shows the XRD spectra of pristine aluminum (Figure 4a), Al/PCPA (Figure 4b), and Al/PCPA/Ag (Figure 4c). Five characteristic diffraction peaks located at 2θ of 38.5° (1 1 1), 44.8° (2 0 0), 65.2° (2 2 0), 78.2° (3 1 1), and 82.5° (2 2 2) can be observed in Figure 4a, which correspond well with peaks in the standard diffraction pattern of face-centered cubic aluminum (JCPDS 04-0787). According to Figure 4b, the diffraction pattern of Al/PCPA is similar to that of pristine aluminum, which indicates the negligible effect of the PCPA layer on the crystalline structure of aluminum microspheres. Other than the diffraction peaks attributed to aluminum, there are five additional characteristic diffraction peaks (as observed in Figure 4c) located at 2θ values of 38.2°, 44.4°, 64.6°, 77.4°, and 81.6°, which correspond to (1 1 1), (2 0 0), (2 2 0), (3 1 1), and (2 2 2) planes of face-centered cubic silver (JCPDS 04-0783), respectively. No diffraction peaks corresponding to silver halide or silver oxide are observed, which clearly demonstrates the purity of the silver generated on the surface of the aluminum microspheres.

**Figure 5:**
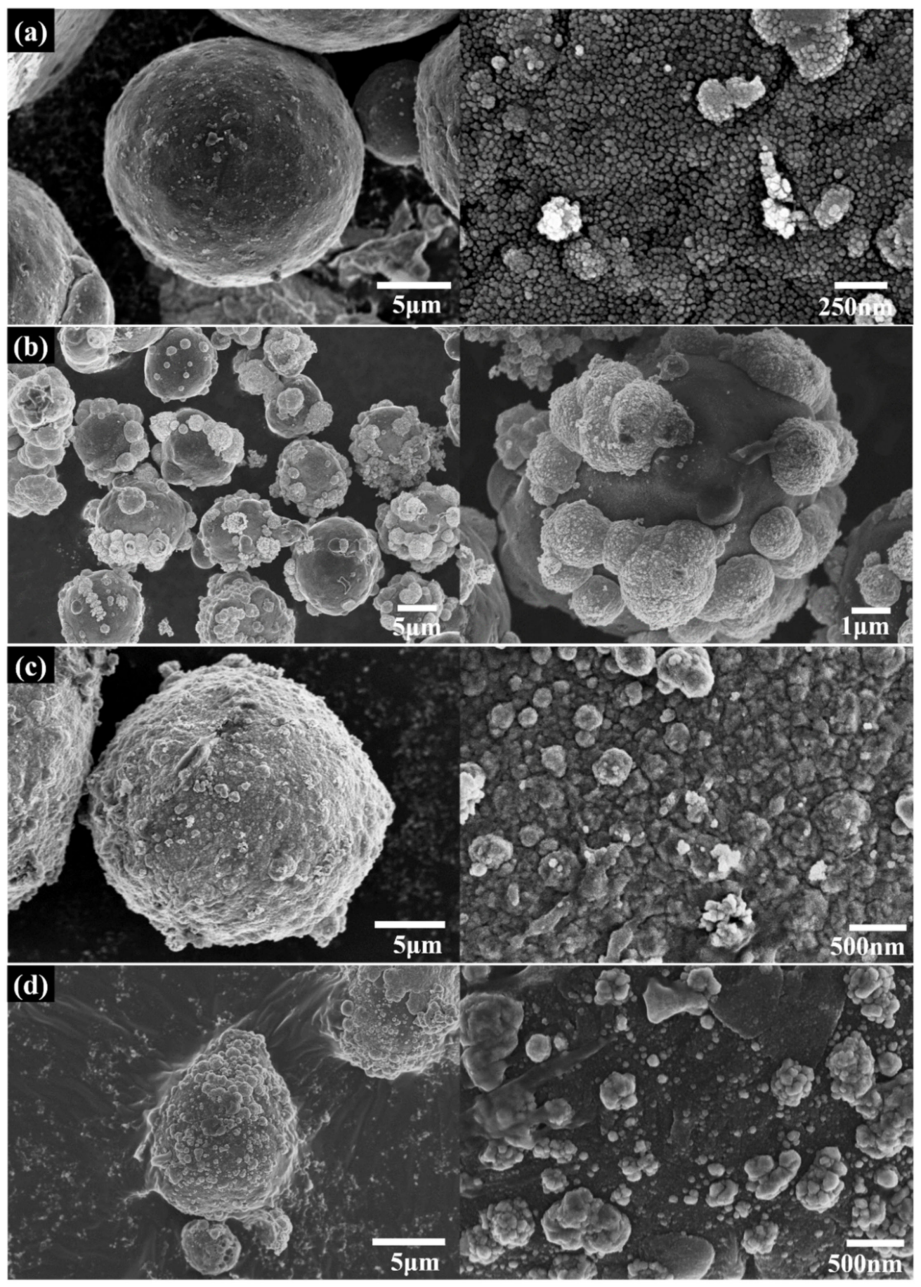
SEM images of Al/PCPA/Ag fabricated with (**a**) EG/OA only (Step 1), (**b**) glucose only (Step 2), and (**c**) a two-step process. SEM images of (**d**) pristine Al/Ag.

The surface morphologies of silver-coated aluminum samples prepared using various methods are shown in Figure 5. Silver nanoparticles were uniformly deposited on the surface of aluminum, as shown in Figure 5a. This is due to the fact that EG can form a chelate with Ag^+^, and this can increase the reduction potential of Ag^+^ [27]. As such, Ag^+^ can be more easily reduced to Ag^0^ due to the addition of EG. However, silver nanoparticles were not in good contact with each other, and this can lead to significant contact resistance. Consequently, the as-prepared Al/PCPA/Ag-Step 1 microspheres possessed a poor electrical conductivity of 26 S/cm (Table 4). Figure 5b shows few silver bulges formed on the surface of the microsphere. Since aluminum powder is involved in the preparation of the two-step process in the reaction, which leads to the decomposition of the silver-plating solution, few Ag^+^ ions are reduced to Ag nanoparticles. Due to the small number of nucleation points, silver nanoparticles start to grow into bulges at these limited sites. In contrast, when there is an abundance of silver particles on the surface of the aluminum microsphere acting as nuclei (obtained from Step 1), these silver particles are able to grow uniformly to form a compact and continuous silver layer on the surface of the aluminum microsphere, as shown in Figure 5c. As a result, the as-prepared Al/PCPA/Ag-Two step microspheres offered the best electrical conductivity of 10,000 S/cm among all prepared samples. To investigate the role of the PCPA layer in the silver-plating process, pristine aluminum microspheres were also used in the two-step silver-plating process. As shown in Figure 5d, the quantity of silver particles formed on the surface of the untreated aluminum microsphere was sparse, and this is attributed to the poor adhesion between silver and aluminum. Thus, based on the collective results, the PCPA layer plays an essential role in the fabrication of a silver-coated aluminum microsphere by functioning as the adhesive layer between silver and aluminum.

**Figure 6:**
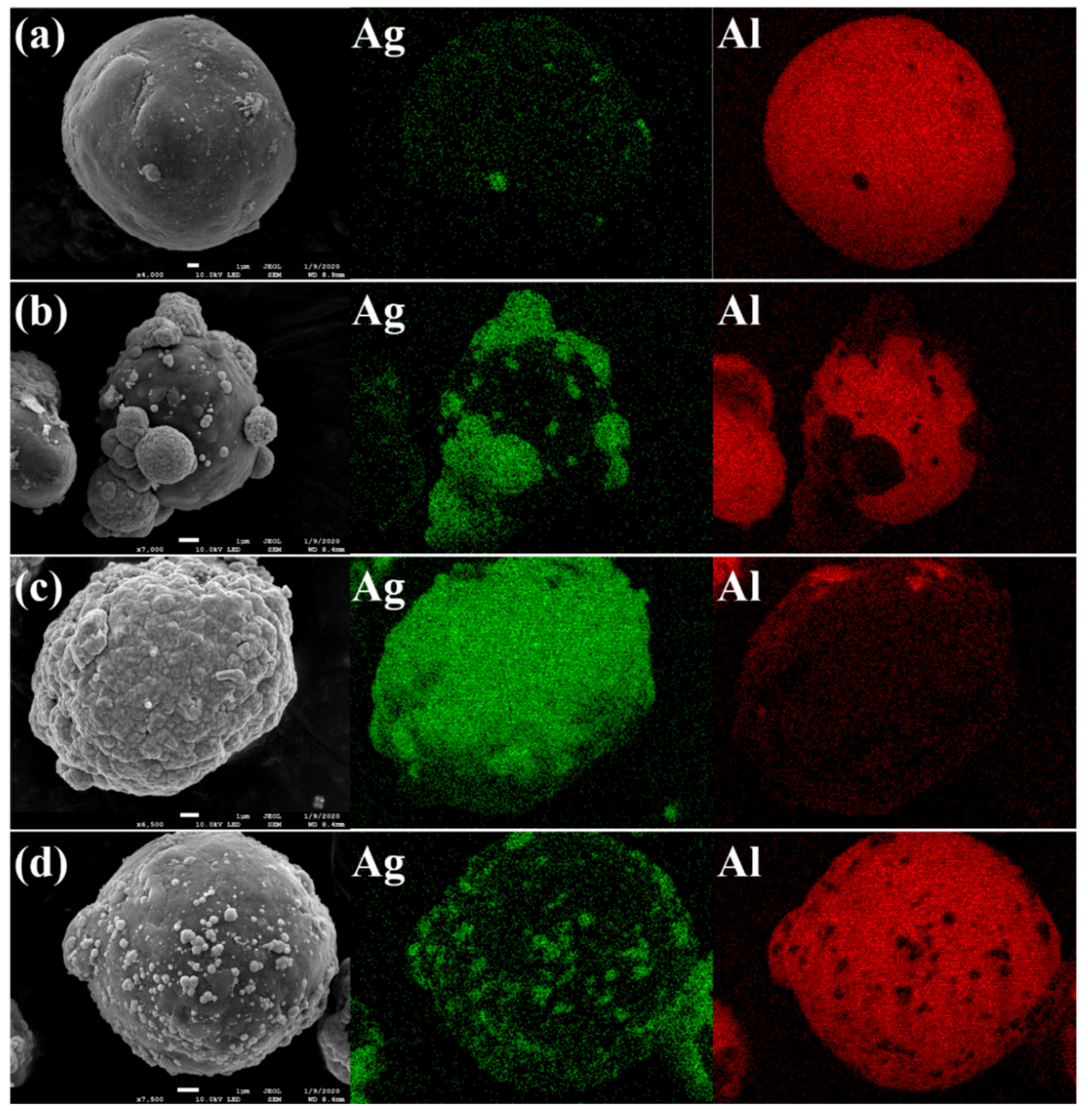
EDX mapping images of (**a**) Al/PCPA/Ag-Step 1, (**b**) Al/PCPA/Ag-Step 2, (**c**)Al/PCPA/Ag two-step, and (**d**) Al/Ag-two step-microspheres.

As it is challenging to distinguish Ag nanoparticles and PCPA coatings based on SEM images, EDX mapping was used to characterize the distribution of silver nanoparticles on the surface of the aluminum microsphere. According to Figure 6, the EDX mapping results are consistent with those in Figure 5. As shown in Figure 6c, a continuous and compact silver layer was coated on the surface of the aluminum microsphere after employing the two-step electroless plating.

**Scheme 3:**
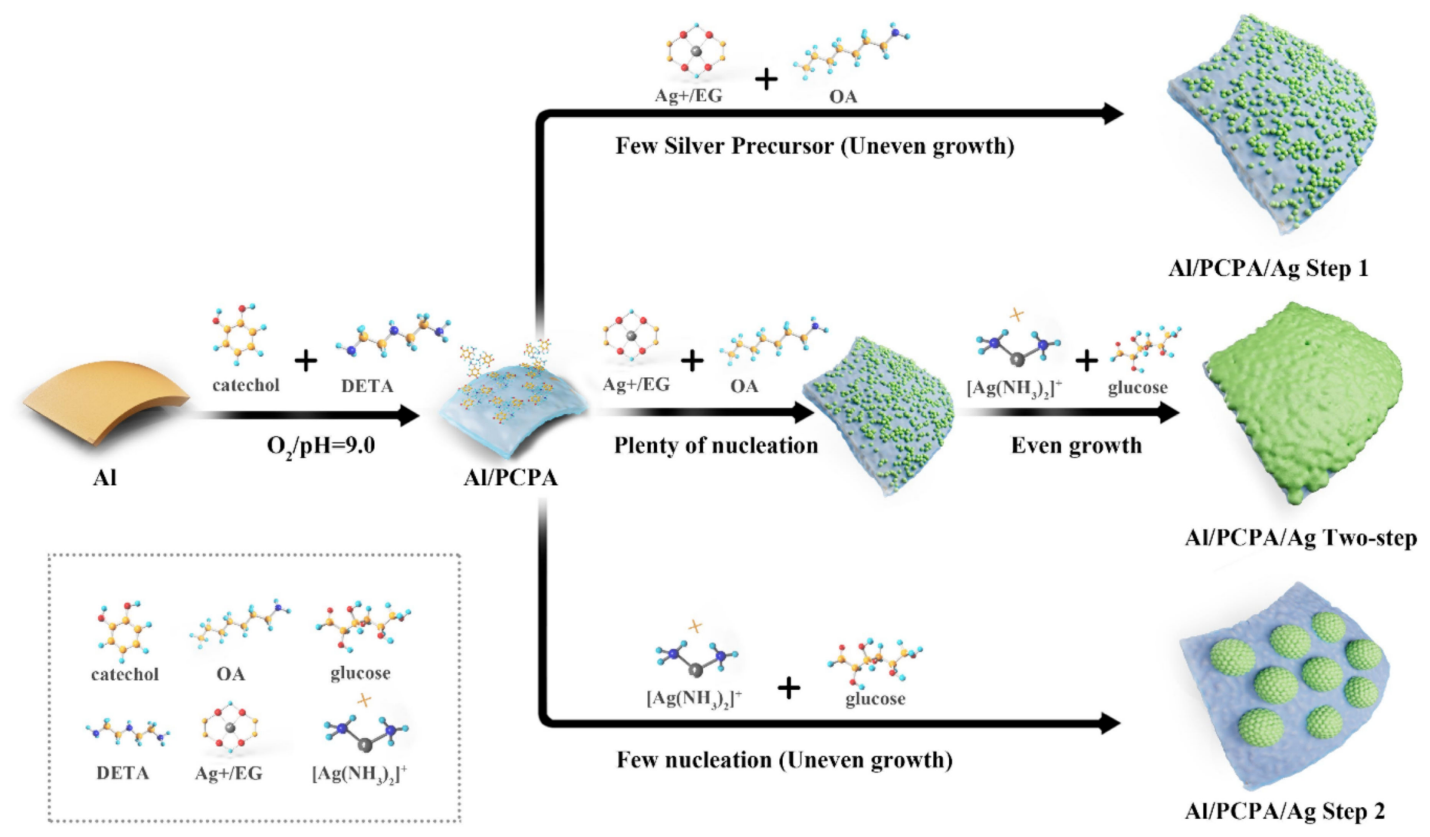
Schematic illustration of the stepwise growth mechanism of Al/PCPA/Ag microspheres.

Scheme 3 shows the stepwise growth mechanism of Al/PCPA/Ag microspheres. After modifying the surface of the aluminum microsphere with PCPA, a large number of silver nanoparticles can be deposited during Step 1 of the electroless plating process. This is because the reduction potential of Ag^+^ is increased by ethylene glycol, and this makes it easy to reduce Ag^+^ to Ag^0^. Despite this advantage, the weak reducing ability of octylamine is insufficient to reduce enough silver nanoparticles to form a continuous silver layer. In contrast, using a relatively strong reducing agent such as glucose can lead to the rapid growth of silver nanoparticles at a few nucleation points, and this, in turn, can cause the formation of bulges on the surface of the aluminum microsphere. Thus, it is necessary to realize a relatively slow growth rate so as to ensure the uniformity and size control of the nanostructure [34]. Moreover, increasing the quantity of nucleation sites is advantageous for the fabrication of a composite particle with a uniform shell. With the abundant silver particles formed during Step 1 that act as nuclei, the silver particles can then grow uniformly to generate a continuous and compact silver layer during the two-step process.

### 3.3. Properties of Al/PCPA/Ag-Filled Silicone Rubber Composite

**Figure 7:**
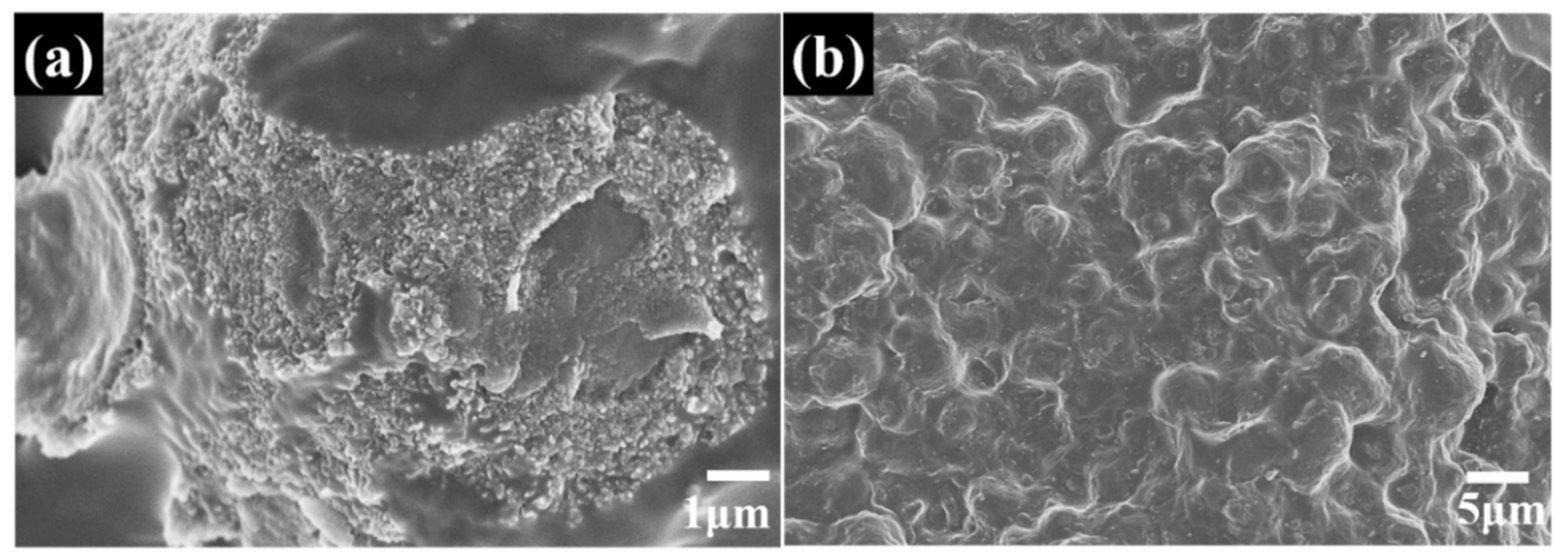
SEM images of Al/PCPA/Ag/MVQ composite (cross-section) under different magnifications (**a**) ×10k, (**b**) ×1k.

**Figure 8:**
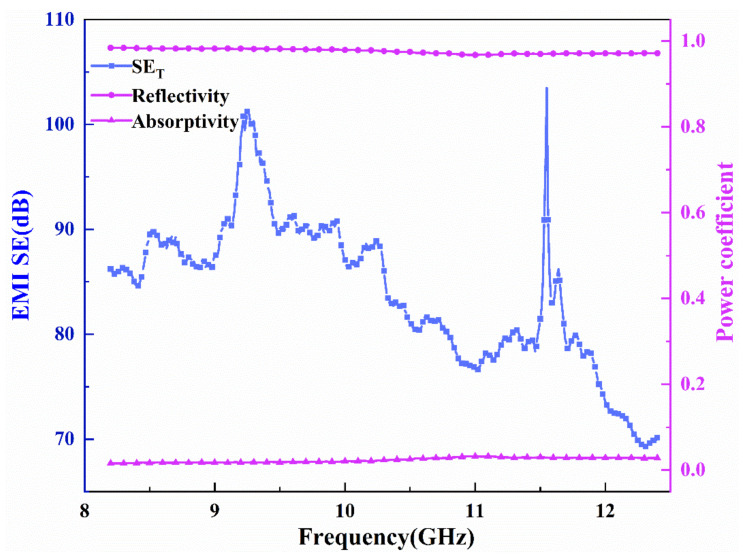
EMI shielding effectiveness and power coefficient of Al/PCPA/Ag/MVQ composite in X-band.

The cross-sectional morphologies of the Al/PCPA/Ag/MVQ composite are shown in Figure 7. It can be observed that the Al/PCPA/Ag microsphere was well incorporated into the MVQ matrix (Figure 7a). The silver layer on the surface of the aluminum microsphere was undamaged, even after the mixing and vulcanization processes, and this indicates that the PCPA layer was able to strongly adhere the silver layer with the aluminum microsphere. Figure 7b shows the even distribution of Al/PCPA/Ag microspheres in the MVQ matrix, without any cavity observed. The Al/PCPA/Ag/MVQ composite filled with 250 phr Al/PCPA/Ag microspheres possessed a low electrical resistivity of 0.0039 Ω·cm. Even after aging at 200 °C × 48 h, the resistivity was still as low as 0.0063 Ω·cm. The EMI SE of Al/PCPA/Ag/MVQ was greater than 70 dB against the X-band with a thickness of 2 cm, whereas the power coefficient of reflectivity (R) was as high as 0.98 (Figure 8). This result means that 98% microwaves are reflected in the propagation process when 99.99999% are attenuated, which is due to the impedance mismatch caused by the ultra-high conductivity of the composite material. This may be promising in applications that require high conductivity and electromagnetic interference shielding.

## 4. Conclusions

A novel approach was developed in this work to fabricate silver-coated aluminum core-shell structured microspheres with high electrical conductivity. Firstly, the oxidative co-deposition of catechol and polyamine was employed to coat PCPA on the surface of an aluminum microsphere. After which, a two-step electroless silver-plating process was proposed to generate silver particles on the surface of an aluminum microsphere. According to the SEM results, the sample prepared using this approach possessed a uniform, compact, and continuous silver shell. Consequently, the as-fabricated Al/PCPA/Ag microspheres possessed a high electrical conductivity of 10,000 S/cm. Furthermore, the Al/PCPA/Ag/MVQ composite demonstrated a low electrical resistivity of 0.0039 Ω·cm, and an EMI SE greater than 70 dB. Thus, the as-fabricated Al/PCPA/Ag/MVQ composite may be considered as a promising conductive and electromagnetic shielding material. Such a method can also be used to deposit silver on other substrates, and it exhibits significant potential in the fabrication of highly conductive particles.

## Figures and Tables

**Table 1:** Formulation of the Al/PCPA/Ag microspheres/rubber composite.

Materials	Dosage (phr)
methyl vinyl silicone rubber (MVQ)	100
Al/PCPA/Ag	250
2,5-Dimethyl-2,5-di(tert-butylperoxy)hexane	2

**Table 2:** Elemental content percentage of pristine aluminum and Al/PCPA microsphere.

Sample	Element (Atomic%)			
	C1s	O1s	Al2p	N1s
pristine Al	30.80	46.73	22.47	-
Al/PCPA	57.36	36.83	1.41	4.40

**Table 3:** Percentage content for carbon functionalities of pristine aluminum and Al/PCPA microsphere.

Sample	Carbon Functionalities (%)			
	C-C & C-H	C=O & C=N	C-O	C-N
pristine Al	86.6	13.4	-	-
Al/PCPA	67.6	6.9	8.3	17.2

**Table 4:** Electrical conductivity of Al/Ag, prepared based on several processes.

Sample	Al/PCPA/Ag-Step 1	Al/PCPA/Ag-Step 2	Al/PCPA/Ag-Two-Step	Al/Ag-Two-Step
Electrical conductivity(S/cm)	26	55	10,000	33

## Data Availability

Not applicable.
